# Healthcare Expenditure and COVID-19 in Europe: Correlation, Entropy, and Functional Data Analysis-Based Prediction of Hospitalizations and ICU Admissions

**DOI:** 10.3390/e27090962

**Published:** 2025-09-16

**Authors:** Patrycja Hęćka, Wiktor Ejsmont, Marek Biernacki

**Affiliations:** 1 Department of Telecommunications and Teleinformatics, Wrocław University of Science and Technology, Wybrzeże Wyspiańskiego 27, 50-370 Wrocław, Poland; wiktor.ejsmont@gmail.com; 2 Department of Mathematics and Cybernetics, Wroclaw University of Economics and Business, Komandorska st. 118/120, 53-345 Wrocław, Poland; marek.biernacki@ue.wroc.pl

**Keywords:** COVID-19, Pearson correlation coefficient, smooth functions, FDA, prediction, Shannon entropy

## Abstract

This article aims to analyze the correlation between healthcare expenditure per capita in 2021 and the sum of the number of hospitalized patients, ICU admissions, confirmed COVID-19 cases, and deaths in a selected period of time. The analysis covers 2017 (before the pandemic), 2021 (during the pandemic), and 2022/2023 (the initial post-pandemic recovery period). To assess the variability and stability of pandemic dynamics across countries, we compute Shannon entropy for hospitalization and ICU admission data. Additionally, we examine functional data on hospitalizations, ICU patients, confirmed cases, and deaths during a selected period of the COVID-19 pandemic in several European countries. To achieve this, we transform the data into smooth functions and apply principal component analysis along with a multiple function-on-function linear regression model to predict the number of hospitalizations and ICU patients.

## 1. Introduction

The SARS-CoV-2 virus emerged in China at the end of 2019 and rapidly escalated into a global crisis. The third wave of COVID-19 in Europe occurred mainly between winter and spring 2021, roughly from February to May 2021. The surge in infections was driven by the emergence of more contagious variants.

The peak of the third wave occurred in March and April 2021, prompting many countries to reintroduce restrictions, including lockdowns, curfews, and travel restrictions. Only with the acceleration of mass vaccinations in the spring of 2021 did the situation begin to improve gradually.

An epidemic of this magnitude inevitably attracts significant attention from researchers and analysts. Numerous studies have examined the impact of the pandemic in Europe, including France [1], Italy [2], Spain [3], and Poland [4,5]. Various methods have been used to study the COVID-19 pandemic, including time series modeling [6], probabilistic models [7], machine learning [8], and deep learning [9]. Cao and Liu [10] compiled a comprehensive overview of these approaches and their applications in analyzing the impact of the pandemic. In recent years, several high-impact studies, including those published in Lancet Regional Health and Nature Medicine (2023–2025), have investigated COVID-19 [11,12,13,14,15].

According to OECD analyses [16], all national healthcare systems were managed inefficiently and ineffectively. It is essential to recall the Council of the European Union’s resolution 2011/C, which “emphasises the fundamental importance of the effectiveness of investments in future health systems, which should be measured and monitored by the relevant Member States”, while “a high level of quality of human health protection should be ensured while maintaining the principles: universality, accessibility, justice and solidarity” [https://eur-lex.europa.eu/LexUriServ/LexUriServ.do?uri=OJ:C:2011:202:0010:0012:PL:PDF (accessed on 24 June 2025)]. Almeida [17], analyzing 173 regions of Europe, showed, among other things, that regions with higher health system efficiency index values in 2017 had significantly higher rates of COVID-19 deaths in 2020 and 2021, suggesting the existence of a trade-off between health system efficiency and health system resilience during the COVID-19 pandemic. In poorer regions or countries, the high efficiency of health systems is a consequence of their underfunding. Similar conclusions were drawn by Coccia and Benati [18]. Analyzing European countries, they showed that a lower COVID-19 mortality rate was strongly correlated with higher per capita healthcare expenditures, as well as a higher number of medical personnel and intensive care beds per 1,000 inhabitants. Therefore, underfunded regional health systems faced greater challenges in treating patients during the pandemic. This naturally led to the question of optimal management of national healthcare systems during the pandemic in European countries.

In this study, we combine correlation analysis, Shannon entropy, and functional data analysis (FDA) to provide a comprehensive assessment of the COVID-19 dynamics across European countries. While correlation analysis identifies dependencies between key variables such as hospitalizations and healthcare expenditure, Shannon entropy quantifies temporal variability and uncertainty, and FDA models entire trajectories over time rather than individual points. This integrated approach allows us to capture not only absolute trends but also variability, stability, and temporal relationships in the data. Compared to previous studies that rely primarily on traditional epidemiological models or simple correlation analyses, our methodology offers novel insights into cross-country epidemic dynamics.

## 2. Materials and Methods

In this article, we analyze a selected period of the pandemic, which includes the third wave of the pandemic in most of the analyzed countries. That wave of the COVID-19 pandemic began in January or February 2021. During this time, many Central European countries, including Poland, the Czech Republic, Hungary, and Slovakia, experienced a sharp rise in infections, driven largely by the spread of more transmissible variants of the virus. In response, further restrictions were implemented and vaccination efforts were accelerated to stop the increase. The third wave began to subside in May 2021, as the effects of widespread vaccinations, ongoing restrictions, and increased population immunity led to a significant decline in new cases. Therefore, in this article, we analyze the period from 7 February to 31 May 2021. The study period from February to May 2021 was chosen because it corresponds to the third wave of the COVID-19 pandemic in Europe, which was characterized by a significant number of hospitalizations and ICU admissions. This period provides a complete and consistent set of daily data across all selected countries, allowing us to analyze the full trajectory of the epidemic wave.

For our analysis, we select countries located in Europe: Austria, Czech Republic, France, Italy, Portugal, Slovakia, Slovenia, Spain, Sweden, and Switzerland.

First, we analyze data on the total annual healthcare expenditure per capita (including both public and private spending) for our selected countries [source: https://ourworldindata.org/grapher/annual-healthcare-expenditure-per-capita, accessed on 5 June 2025; https://data.worldbank.org/indicator/SH.XPD.CHEX.PC.CD, accessed on 18 June 2025] in 2017, 2021, and 2022 or 2023. Then, we calculate the Pearson correlation coefficient, along with *p*-values and 95% confidence intervals, between health expenditure per capita in 2021 and sum of the number of hospitalized patients, ICU admissions, confirmed COVID-19 cases, and deaths from 7 February to 31 May 2021. The next step in the article is to include three variables separately: the number of COVID-19 deaths, confirmed cases, and healthcare expenditure in Poland, and then perform a correlation analysis involving these new variables. Then, to assess the variability and stability of pandemic dynamics across countries, we compute Shannon entropy for hospitalization and ICU admission data.

The second aim of this article is to predict the number of hospitalized and ICU patients during the chosen time of the COVID-19 pandemic in selected European countries. We test our model on two selected countries: Czech Republic and Italy. The remaining countries will serve as the training set. The countries included in this study were selected based on three criteria: (1) location in Europe with diverse healthcare systems and population sizes, (2) availability of daily data on infections, hospitalizations, ICU patients, and deaths for the period 7 February to 31 May 2021, and (3) representation of varying epidemic dynamics to ensure a diverse training dataset. Data from the Czech Republic and Italy were reserved as the test set, while the remaining countries were used for training. This approach allows evaluation of the model’s ability to generalize to countries not included in the training set. However, the results are most representative of European countries with similar reporting systems and epidemic trends and may not fully generalize to countries outside Europe or with different healthcare and reporting practices.

In addition, an analysis of functional principal components is performed. The results in this study are obtained using R software, specifically the packages ‘ggplot2’ https://CRAN.R-project.org/package=ggplot2 and ‘fda’ https://CRAN.R-project.org/package=fda (accessed on 15 February 2025). The observed data consist of the daily cumulative reported values of these four functional variables per million people for the ten countries, covering the period from 7 February 2021 to 31 May 2021 (see data source https://ourworldindata.org/coronavirus, accessed on 15 February 2025).

## 3. Health Expenditure per Capita

**Figure 1:**
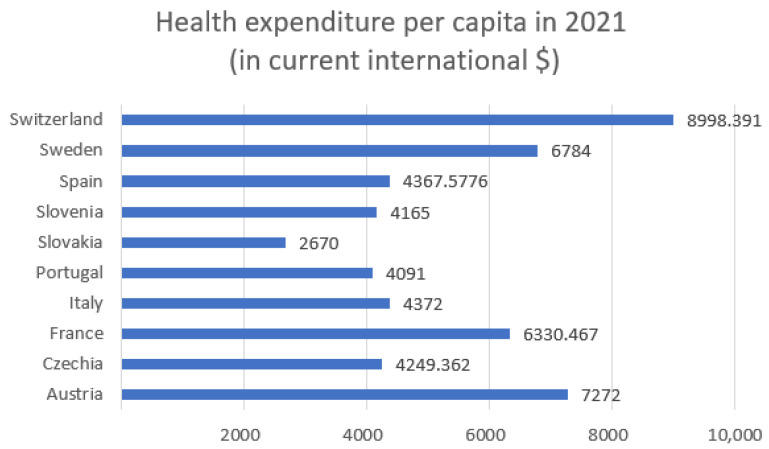
Health expenditure per capita in 2021. This data is expressed in current international dollars.

**Figure 2:**
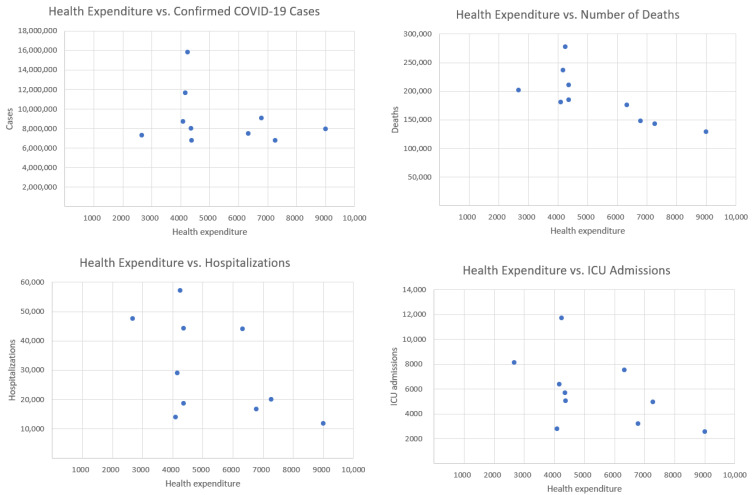
Health expenditure per capita (in current international dollars) in 2021 and four COVID-19 indicators.

In this section, we analyze the correlation between total health expenditure per capita in 2021 and the sum of the number of daily observations of hospitalized patients, ICU admissions, confirmed COVID-19 cases, and deaths from 7 February to 31 May. Figure 1 shows the health expenditure per capita in 2021 for selected European countries and Figure 2—health expenditure and our four COVID-19 indicators. Table 1 presents the Pearson correlation coefficients between total healthcare expenditure per capita and four key COVID-19 indicators across selected European countries, along with *p*-values and 95% confidence intervals. Among the variables considered, only the correlation with deaths is statistically significant (*r* = −0.734, *p* = 0.016), indicating a strong negative association. The correlations with confirmed cases, hospitalizations, and ICU admissions are not statistically significant (*p* > 0.1), with wide confidence intervals reflecting the limited sample size (*n* = 10).

The correlation with confirmed cases is weak and negative (r=−0.2506), suggesting that countries with higher healthcare spending tend to have slightly fewer confirmed cases. However, this relationship is weak and may be influenced by other factors, such as testing policies or population density. The strongest negative correlation is observed between health expenditure and deaths (r=−0.7341). This indicates a strong inverse relationship: countries that spend more on healthcare tend to report significantly fewer COVID-19-related deaths. This finding suggests that better-funded health systems may be more effective in preventing fatal outcomes. The correlation with hospitalizations is moderately negative (r=−0.5217), implying that increased healthcare investment may contribute to reducing the burden on hospitals during the pandemic. Similarly, there is a moderate negative correlation between health expenditure and the number of ICU admissions (r=−0.4974). This suggests that more robust healthcare systems might mitigate the severity of cases requiring intensive care. Overall, the analysis reveals a consistent negative relationship between healthcare spending and COVID-19 impact. While the correlation with case numbers is weak (likely due to external factors), the stronger associations with hospitalizations, ICU admissions, and especially deaths suggest that higher investment in healthcare correlates with better pandemic outcomes, particularly in reducing mortality.

## 4. Health Expenditure per Capita with Addition of Poland

**Figure 3:**
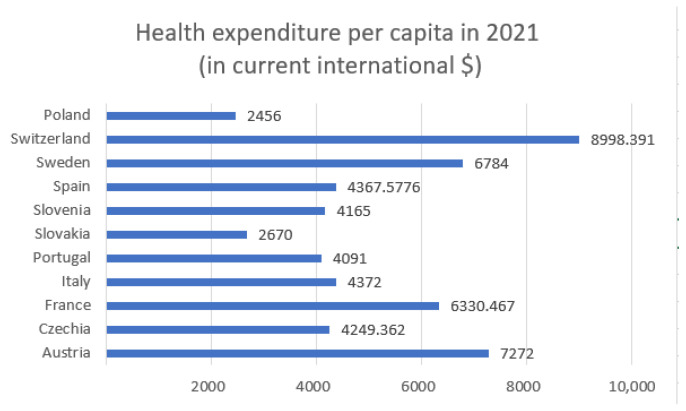
Health expenditure per capita in 2021, with addition of Poland. This data is expressed in current international dollars.

**Figure 4:**
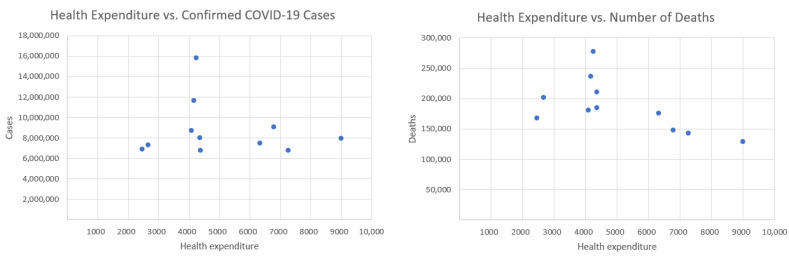
Health expenditure per capita (in current international dollars) in 2021, with addition of Poland and two COVID-19 indicators.

In this section, we compute the correlation coefficient between health expenditure per capita and the cumulative number of daily confirmed COVID-19 cases and deaths, covering the period from 7 February to 31 May, with the addition of Poland. Due to data limitations, variables related to hospitalizations and ICU admissions are excluded from the analysis. Figure 3 and Figure 4 display health expenditure per capita for selected European countries, as in Figure 1 and Figure 2, but with the addition of Poland. Figure 4 presents health expenditure and our two COVID-19 indicators. Table 2 presents the Pearson correlation coefficients, along with *p*-values and *t*-statistics, between total healthcare expenditure per capita and two key COVID-19 indicators across selected European countries.

The inclusion of Poland’s health expenditure data resulted in a lower correlation with both COVID-19 deaths and confirmed cases.

Initially, the correlation between healthcare expenditures and mortality was −0.734, indicating a strong negative relationship—suggesting that higher healthcare spending is associated with lower death rates.

However, after introducing an additional variable indicating whether the data point corresponds to Poland, the correlation dropped to −0.592. This change has important implications for interpreting the data. Poland has relatively low healthcare spending and quite high mortality, which differ from other countries in the dataset. These differences can distort the perceived relationship between healthcare expenditures and deaths. Although the strength of the correlation has decreased, it remains moderately strong and negative. This implies that higher healthcare spending still tends to be associated with lower mortality—but the effect is less pronounced. The *p*-value is equal to 0.054. The correlation is marginally non-significant at the conventional 0.05 level, suggesting a possible trend where higher health expenditure may be associated with fewer deaths, but the evidence is not conclusive.

Initially, the correlation between the number of COVID-19 cases and healthcare expenditure was −0.2506, indicating a weak negative relationship—suggesting that, to a small extent, higher healthcare spending might be associated with fewer COVID-19 cases. After introducing a new variable where the data point corresponds to Poland, this correlation further decreased to −0.1218, making the relationship even weaker. With a correlation of just −0.1218, the relationship is very weak and likely not statistically significant. This means there is little to no meaningful linear association between healthcare expenditures and COVID-19 case counts when country effects like those of Poland are taken into account. The observed decrease in correlation suggests that healthcare spending has a limited and possibly indirect role in influencing COVID-19 case numbers. Other country-specific factors likely drive case rates more strongly, and including these in the analysis helps to avoid overestimating the impact of healthcare investment alone. The *p*-value is equal to 0.72. This indicates no statistically significant relationship between health expenditure and the number of confirmed cases.

## 5. Health Expenditure per Capita in 2017 and 2022/2023

**Figure 5:**
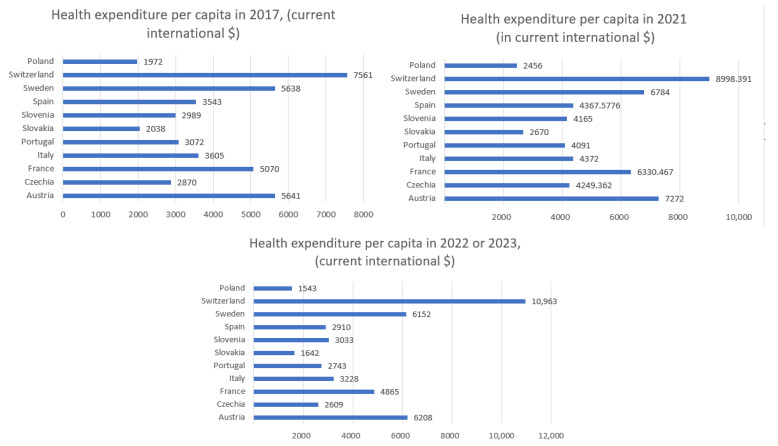
Health expenditure per capita (in current international dollars) in 2017, 2021, and 2022/23.

In this section, we calculate the correlation coefficient between health expenditure per capita in 2017 (prior to COVID-19) and in 2022/23, and the cumulative numbers of daily confirmed COVID-19 cases and deaths recorded between 7 February and 31 May. Figure 5 displays health expenditure per capita for selected European countries in 2017 and in 2022 or 2023. Owing to data gaps, data for some countries pertain to 2022, whereas, for a few others, data from 2023 are available.

The presented data in Figure 5 illustrate the dynamics of health expenditure per capita before, during, and after the main phase of the COVID-19 pandemic. In 2021, most countries experienced a noticeable increase in health spending, likely driven by pandemic-related needs such as treatment, testing, and vaccination programs. In 2022/2023, a general stabilization or even reduction in health expenditure is observed, reflecting the gradual normalization of healthcare systems. However, significant disparities between countries persist, with wealthier nations maintaining substantially higher per capita spending compared to Central and Eastern European countries.

Table 3 and Table 4 present Pearson’s correlation coefficients between total health expenditure per capita and two COVID-19 outcome variables: cumulative deaths and confirmed positive cases across three reference years—2017 (pre-pandemic baseline), 2021 (during the height of the pandemic), and 2023 (post-acute phase of the pandemic), along with *t*-statistics and *p*-values.

Across all three time points, the results consistently indicate a negative correlation between health expenditure per capita and both COVID-19 deaths and confirmed cases. However, the strength and implications of these correlations vary between the two outcome variables. The negative correlations between health spending and COVID-19-related deaths are relatively strong and stable across time. These values suggest that countries with higher per capita health expenditures tended to experience lower COVID-19 mortality rates regardless of the pandemic phase. The fact that the strength of this association persists across pre-pandemic and post-pandemic years implies that investment in healthcare infrastructure, accessibility, and quality likely played a significant role in reducing fatal outcomes. In contrast, the correlation between health expenditure and the number of positive COVID-19 cases is consistently negative but weaker, with coefficients ranging from −0.1218 to −0.2111. Table 3 shows strong correlations in 2017 (*r* = −0.658; *p* = 0.028) and 2023 (*r* = −0.639; *p* = 0.036), both statistically significant at the 0.05 level. The 2021 correlation (*r* = −0.592) is marginally non-significant (*p* = 0.054). These results suggest a trend where higher health expenditure may be associated with fewer COVID-19 deaths, although the small sample size (*n* = 11) limits the certainty of this conclusion. The correlations between health expenditure and the number of confirmed COVID-19 cases are not statistically significant (*p*-values from 0.53 to 0.72). This indicates no evidence of a significant relationship between healthcare expenditure per capita and the number of confirmed cases in this dataset.

Table 5 shows negative correlations between health expenditure per capita and both COVID-19-related hospitalizations and intensive care unit (ICU) admissions across three time points: before the pandemic (2017), during the pandemic peak (2021), and post-acute phase (2022). Poland is excluded from this analysis because of missing data.

In all periods, higher health expenditure per capita is associated with lower numbers of hospitalizations and ICU admissions. This suggests that stronger healthcare systems may be better equipped to manage patients earlier, preventing severe disease progression that requires hospitalization or intensive care. The strength of correlations remains relatively stable across years for both hospitalizations (around −0.52 to −0.54) and ICU cases (around −0.49 to −0.55). This stability may reflect that, regardless of the pandemic phase, health system capacity plays a consistent role in mitigating severe clinical outcomes. Both sets of results suggest a negative relationship between health expenditure per capita and severe COVID-19 outcomes (hospitalizations and ICU cases), but the associations are not statistically significant. The limited sample size (*n*= 10) reduces statistical power, so conclusions should be drawn cautiously.

## 6. Shannon Entropy of COVID-19 Hospitalizations and ICU Admissions

In this section, we calculate the Shannon entropy of COVID-19-related hospitalizations and ICU admissions for selected countries. Table 6 and Table 7 present the entropy values based on rounded daily counts of confirmed hospitalizations and ICU admissions.

Czechia (6.69) and Slovakia (6.65) show the highest entropy in hospitalizations, indicating high variability and unpredictability in the number of hospital admissions over time. Italy (6.44) and Austria (6.34) also exhibit elevated entropy, consistent with complex or fluctuating hospitalization dynamics. On the other end, Sweden (5.63) and Switzerland (5.47) had the most consistent patterns, suggesting either more controlled surges or smoother hospital capacity management.

Also, Czechia (6.15) and Slovakia (5.87) lead with the highest ICU entropy, implying that critical care demand was highly variable, perhaps due to rapidly evolving case severity or resource strain. Switzerland (3.60) and Sweden (4.00) show the lowest entropy in ICU data, suggesting a more stable or regulated use of ICU beds, potentially due to early triage policies or fewer severe cases.

In the analysis of the entropy of hospitalizations and ICU admissions in European countries, it is also important to consider healthcare expenditure per capita, which may influence the capacity of healthcare systems to respond effectively to a pandemic.

The highest expenditures (Figure 1) were recorded in Switzerland (approximately USD 8998), Austria (USD 7272), and Sweden (USD 6784). Countries such as Slovakia (USD 2670), and Slovenia (USD 4165) reported significantly lower per capita health spending. Switzerland stands out with the lowest entropy in both hospitalizations (5.47) and ICU admissions (3.60) while also having the highest health expenditure. This may suggest that a well-funded healthcare system contributes to more stable and predictable patterns in hospital and intensive care usage during a pandemic. Conversely, countries with higher entropy, such as Czechia (hospitalizations: 6.69; ICU: 6.15) and Slovakia (hospitalizations: 6.65; ICU: 5.87), have relatively lower health expenditure per capita (approximately USD 4249 and USD 2670, respectively). This could indicate greater challenges in maintaining stability within the healthcare system and responding efficiently to the fluctuating demands of a pandemic. Countries with moderate levels of expenditure, such as Italy (USD 4372) and France (USD 6330), demonstrate intermediate entropy values for hospitalizations and ICU admissions, which may reflect a moderate degree of variability and some resilience within their healthcare systems. Poland, which had the lowest healthcare expenditure among the analyzed countries, was not included in the entropy analysis. However, based on the available data, it could be assumed that limited financial resources may contribute to higher variability and difficulties in managing hospital capacity during pandemic waves.

Higher per capita health expenditure may promote more stable and predictable trends in hospitalizations and ICU admissions, as reflected in lower entropy values. In contrast, countries with lower health investments may face increased entropy, indicating greater difficulty in managing pandemic surges, bed availability, and implementing consistent health policies. Recognizing this relationship underscores the importance of investing in healthcare systems as a key factor in building resilience against future health crises.

## 7. Principal Component Analysis

In this section, we apply principal component analysis (PCA) for functional data to examine the training group of the COVID-19 data. The concept of PCA in the functional framework was extensively discussed by Ramsay and Silverman ([19], Section 8). In functional PCA, each component represents a dominant mode of variation among epidemic curves. The first component generally corresponds to overall intensity, distinguishing countries with higher versus lower hospitalization levels. The second component often captures differences in timing or shape, such as the speed of increase or the location of the epidemic peak. Higher-order components describe finer details, such as variations in the duration or amplitude of epidemic waves. Let us denote by $X_{1}\left(t\right),X_{2}\left(t\right),Y_{1}\left(t\right),Y_{2}\left(t\right)$ the time-dependent variability of the following variables: number of cases, deaths, hospitalized, and intensive care patients, respectively. The first principal components explain approximately 98%,94%,74%,66% of the variability in $X_{1}\left(t\right),X_{2}\left(t\right),Y_{1}\left(t\right),Y_{2}\left(t\right)$, respectively, for the countries included in the training sample. A high variance captured by the first component suggests that a large portion of the variation in COVID-19 outcomes, such as cases (98%) and deaths (94 %) across countries or regions, can be summarized by a single dominant mode of variation. In other words, most regions follow a similar dynamic trend over time. Essentially, this component captures the dominant pattern of how cases and deaths evolve over time, highlighting common temporal dynamics across regions.

First, all curves are mapped to the common interval [0, 1], where the original time is denoted as T∈[0,max(T)], any response variable as *z*, and the transformed response variable as $z^{*}$. The value max(T) represents the maximum *T* in all curves, ensuring consistency. The registration equation is then provided by

$$z^{*}\left(u\right)=z\left(u\cdot \max\left(T\right)\right),\;\forall u\in \left[0,1\right].$$

**Figure 6:**
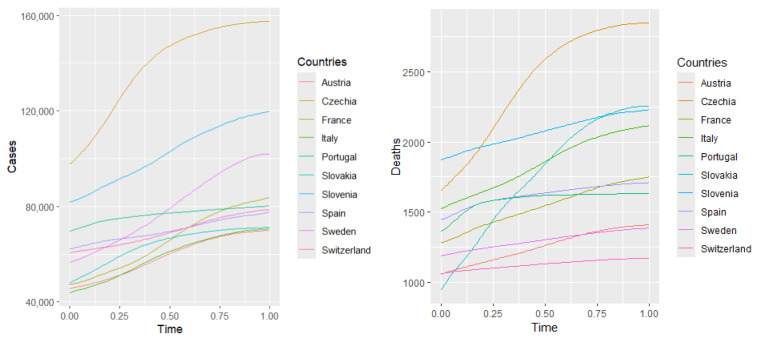

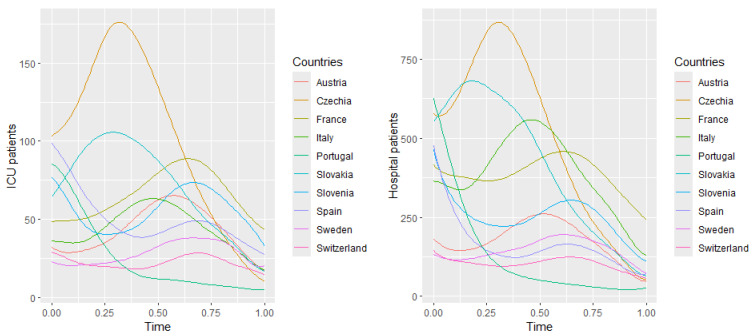
Fitted curves with 10 basis functions applied to daily observations of the number of cases, deaths, hospitalized patients, and ICU patients from 7 February 2021 to 31 May 2021 in selected European countries.

Various methods have been used to convert the data into smooth functions [19,20,21]. Figure 6 illustrates the fitting of 10 basis functions with equally spaced knots to approximate curves representing hospitalizations, ICU patients, confirmed cases, and deaths. The coefficients are estimated using the least squares method ([19], Section 4.2). The number of basis functions is selected to minimize the mean squared error, ensuring an optimal bias–variance trade-off and preventing overfitting.

### 7.1. Mean Curve

**Figure 7:**
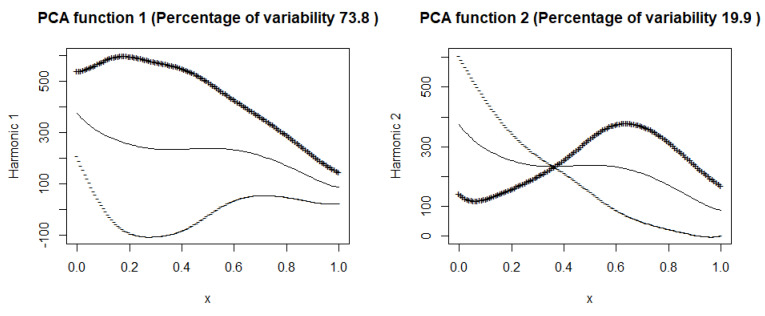
Mean curve of hospitalized patients along with curves obtained by adding (+) and subtracting (–) appropriately scaled harmonic coefficients from the mean.

**Figure 8:**
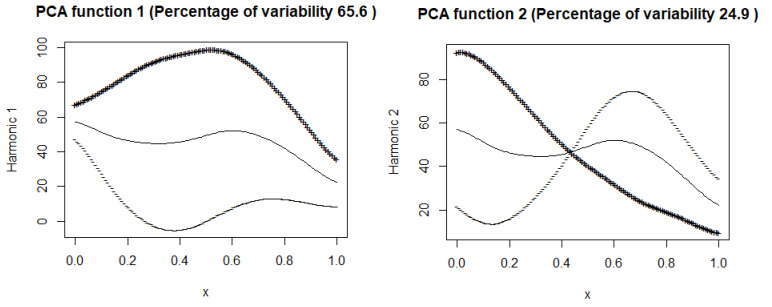
Mean curve of ICU patients along with curves obtained by adding (+) and subtracting (–) appropriately scaled harmonic coefficients from the mean.

This subsection displays plots of the mean curve along with functions obtained by adding and subtracting appropriately scaled weight functions from the mean. Figure 7 and Figure 8 illustrate these plots for the number of hospitalized and intensive care patients. In Figure 7 (hospitalized patients), the first principal component explains 73.8% of the variance, suggesting that this component describes well the variability in the number of hospitalized people. In the Figure 8 (ICU patients), the first component explains 65.6% of the variance, suggesting that, although still dominant, the variability in the ICU patient data is more complex than in the case of hospitalizations. Both harmonic functions have a similar shape, where the values are highest at the beginning and then gradually decrease. In both cases, it can be seen that the number of hospitalized and seriously ill patients is initially high and then gradually decreases. The decrease in both cases suggests the effectiveness of treatment, pandemic restrictions, or increased population immunity.

The second components explain 19.9% and 24.9% of the variance. The figures suggest that the first principal components represent the overall trend in the number of hospitalized people and ICU patients.

### 7.2. Principal Component Scores

**Figure 9:**
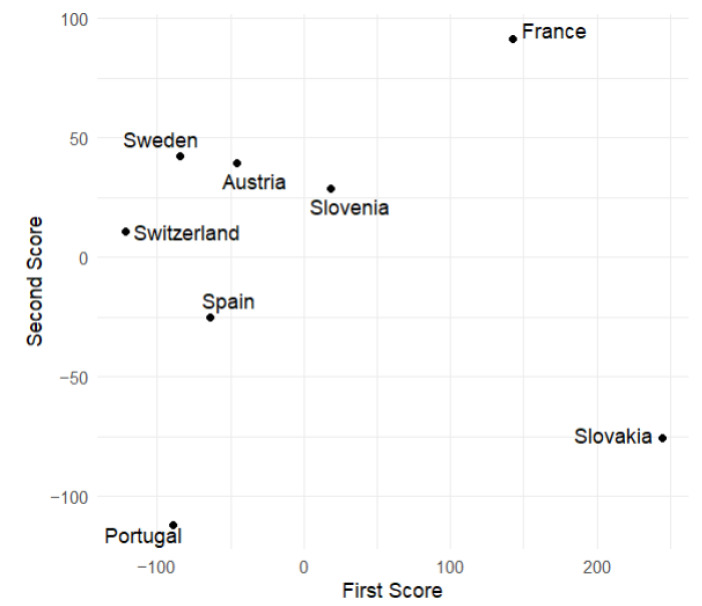
Values of the first and second scores for the number of hospitalized patients during the analyzed time of the pandemic (European countries from the training sample).

**Figure 10:**
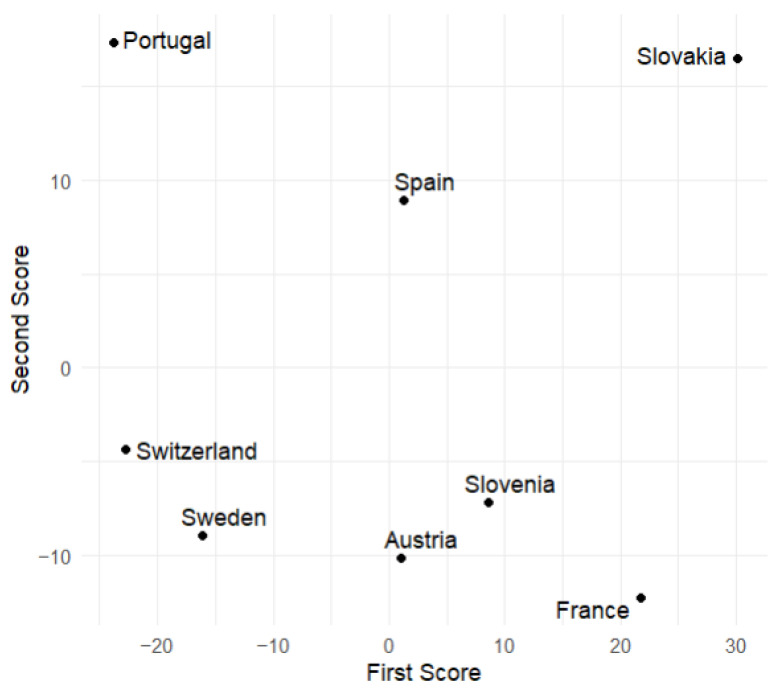
Values of the first and second scores for the number of ICU patients during the analyzed time of the pandemic (European countries from the training sample).

This subsection displays plots of the variable values corresponding to the scores of the first and second principal components. From Figure 9 and Figure 10, we observe that Slovakia and France have the highest numbers of hospitalized patients during the analyzed time of the pandemic regarding the training group, while Switzerland and Portugal have the lowest. This is corroborated by actual data. Figure 6 suggests that, at the beginning of the analyzed time, Slovakia has the highest number of hospitalized and ICU patients among the countries in the training group. In France, the number of hospitalized and ICU patients is higher during the second half of the analyzed time.

## 8. Prediction

In this section, we apply the multiple function-on-function linear model to predict the number of hospitalized individuals and ICU patients during the chosen time of the COVID-19 pandemic in selected European countries. The predictions are based on the multiple function-on-function linear model, as presented in Equation (1). That model was described in detail in [3,22].

(1) $${\hat{y}}_{ik}\left(t\right)={\bar{y}}_{k}\left(t\right)+{\hat{\xi }}_{i1}^{y_{k}}f_{1}^{y_{k}}\left(t\right),\;k=1,2;i=1,\ldots ,10,$$

where ${\bar{y}}_{k}$ is the mean of the number of hospitalized people (for k=1) and the number of ICU patients (for k=2) for countries from the training group (8 countries). Functions $f_{1}^{y_{k}}\left(t\right)$ are the eigenfunctions (weight functions) of the sample covariance. The estimators of the principal component scores for $y_{1}$ and $y_{2}$ are described by Equation (2):

(2) $${\hat{\xi }}_{i1}^{y_{k}}=\gamma_{0}+\xi_{i1}^{x_{1}}\gamma_{1}^{y_{k}}+\xi_{i1}^{x_{2}}\gamma_{2}^{y_{k}}+\epsilon_{i}^{y_{k}},k=1,2,i=1,...,10,$$

where $\gamma_{0},\gamma_{1}^{y_{k}},\gamma_{2}^{y_{k}}$ are the appropriate coefficients obtained by fitting the linear model to the data, and $\epsilon_{1}^{y_{k}}$ are independent functional errors.

### 8.1. Training Sample

**Figure 11:**
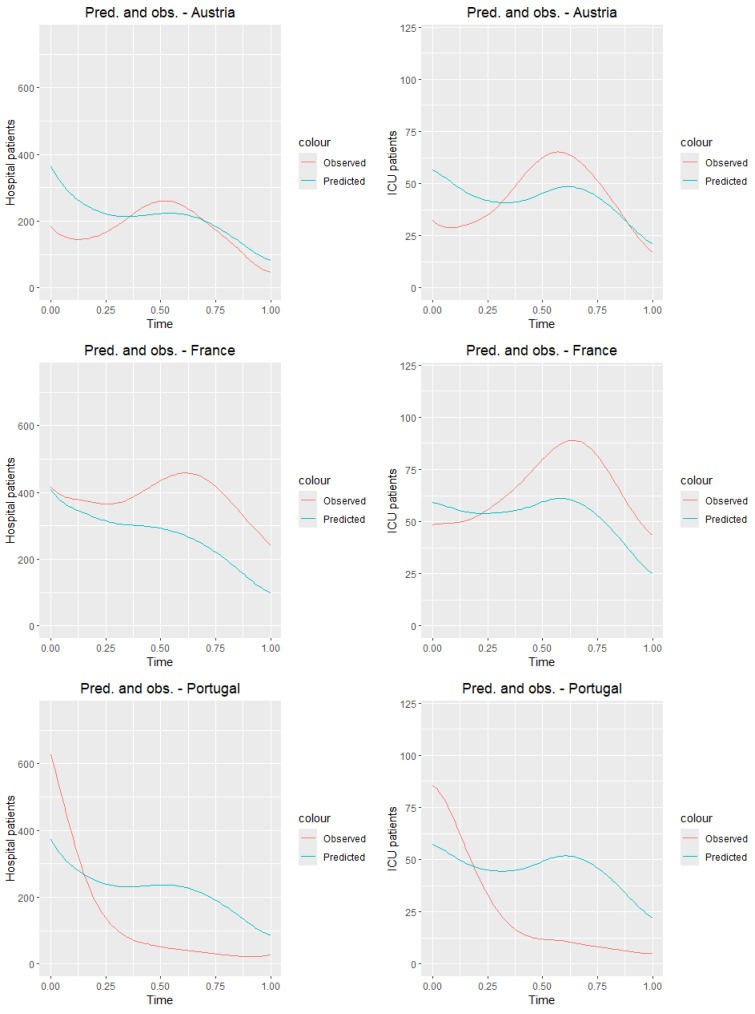

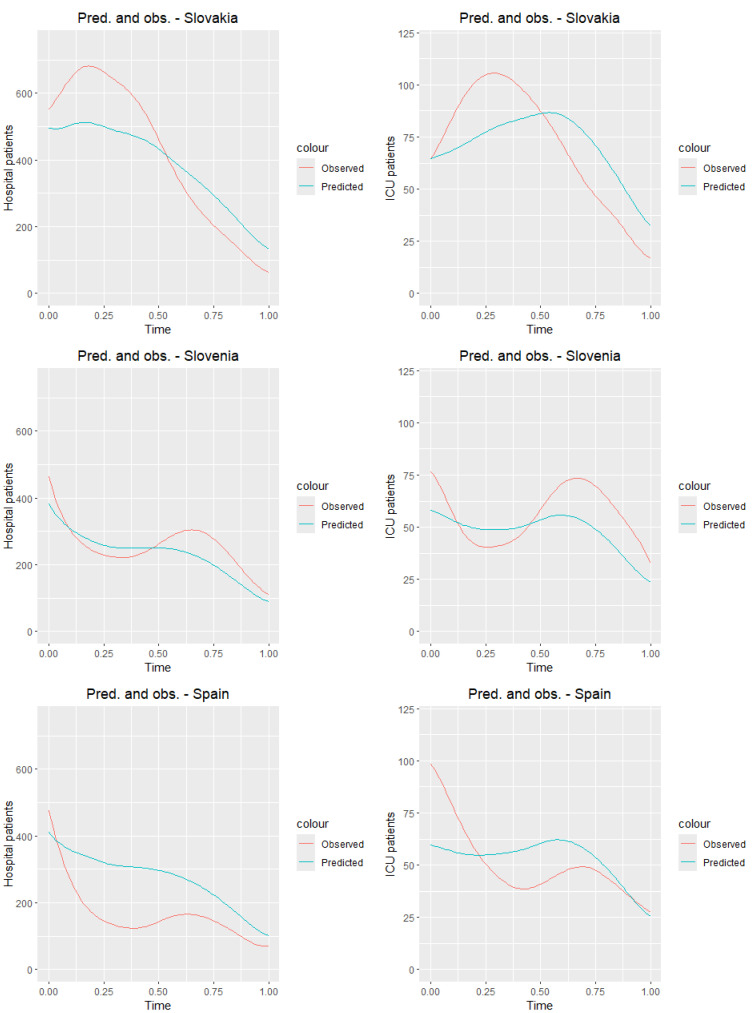

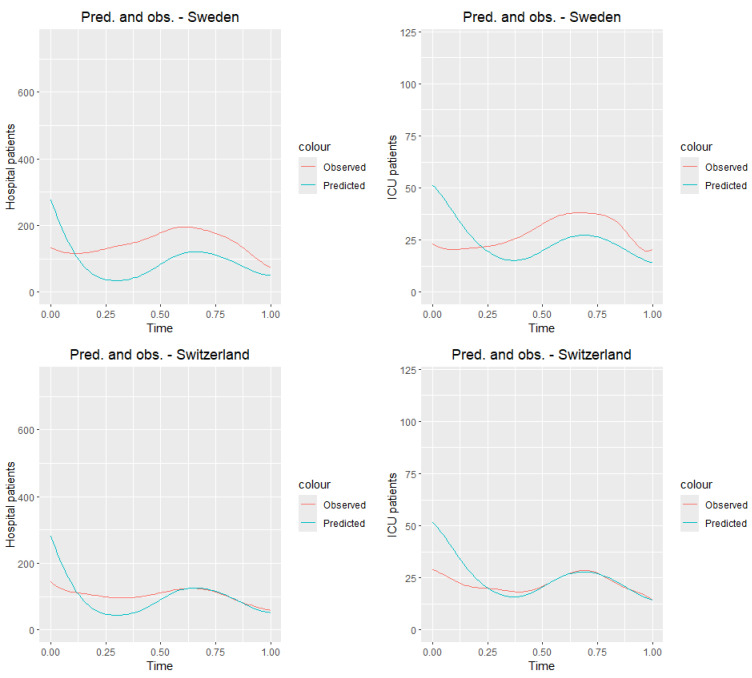
Observed and predicted curves for countries from the training sample.

First, we test the model on the training sample. Figure 11 presents the graphs of the observed curves (Figure 6) along with the graphs of the predicted curves for the countries of the training sample. Table 8 and Table 9 present the values of the mean squared error and root mean squared error for all countries from the training sample. Analyzing tables and Figure 11, we observe that the predictions closest to the true values for both ICU and hospitalized patients are obtained for Switzerland, while the worst results are seen for Portugal. Although the results deviate from the true daily values, it is clear that, for most countries, the prediction graph captures the general trend of the analyzed time of the pandemic.

### 8.2. Test Sample

**Figure 12:**
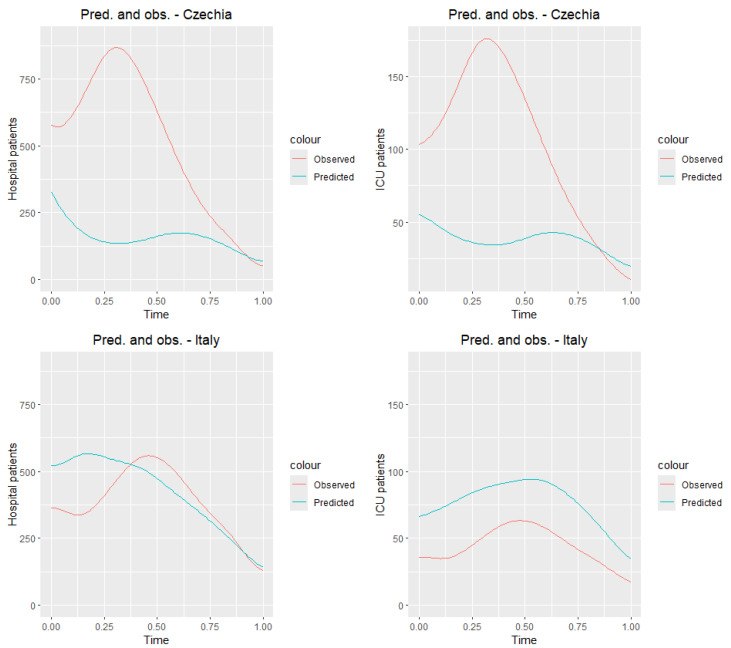
Observed and predicted curves for countries from the testing sample.

Now we test our model in the testing group, Czechia and Italy. Figure 12 presents the observed curves (Figure 6) and predicted. Table 10 and Table 11 show the prediction and daily observation values (see the data source https://ourworldindata.org/coronavirus, accessed on 15 February 2025) for selected dates for countries in the testing group. We receive very good predictions for Italy. Czech Republic is the country where the number of people hospitalized and ICU patients was the highest among the selected countries Figure 6. Hence, we can expect that the reported values are much lower than the real ones.

## 9. Conclusions

The third wave of the COVID-19 pandemic occurred in early 2021, primarily in Europe. It was driven by more transmissible virus variants, particularly the Alpha variant, first detected in the United Kingdom. Hospitalizations and ICU admissions increased, forcing many countries to reintroduce strict restrictions. The vaccination efforts began to accelerate, helping to gradually control the situation and reduce the number of severe cases. In our article, we calculated the Pearson correlation coefficients, along with *p*-values, between total healthcare expenditure per capita and selected COVID-19 indicators in European countries in 2017, 2021, and 2022/2023. Recall the results for 2021. A strong negative correlation is observed with deaths (r=−0.7341;p=0.016), suggesting that higher healthcare spending is associated with fewer fatalities. The correlation with confirmed cases is weak and negative (r=−0.2506;p=0.485), indicating only a slight relationship. Moderate negative correlations were found for hospitalizations (r=−0.5217) and ICU admissions (r=−0.4974), implying that better-funded systems may reduce severe cases. Overall, the results suggest that higher healthcare expenditure tends to correlate with better COVID-19 outcomes, particularly in terms of reducing deaths and severe cases. The data indicate that countries with higher per capita health expenditure generally experienced fewer hospitalizations and ICU admissions due to COVID-19. This highlights the role of healthcare resources not only in treatment capacity but also in early intervention, disease monitoring, and management of comorbidities, which can prevent clinical deterioration. Strong healthcare infrastructure appears to provide resilience in both the acute and post-acute phases of the pandemic.

What do the correlations indicate? The correlation between COVID-19 deaths and health expenditure per capita is strongly negative in every year observed (ranging from −0.59 to −0.66). This suggests that higher health spending per capita is consistently associated with lower mortality. The correlation between positive COVID-19 tests and health spending is weaker (between −0.12 and −0.21), indicating that case detection is less dependent on spending and more influenced by testing policies and public behavior. From the Table 3 of health expenditure and the fitted curves of daily observations of deaths in Figure 6, we can observe that those countries with the highest per capita health spending—such as Switzerland, Sweden, Austria, and France—consistently maintained spending levels above USD 5000–6000 (in current international dollars) and better COVID-19 health outcomes (as indicated by the correlation and other international studies). In contrast, countries like Poland, Slovakia, Slovenia, and Czechia, with spending below this threshold, experienced higher mortality rates. To achieve comparatively better health outcomes during health crises like COVID-19, a minimum health expenditure of USD 5000–6000 per capita (PPP) appears necessary.

It should be noted that the *p*-values for the correlations were calculated using data from only 10–11 countries, which limits the statistical power of the analysis. Consequently, while the reported *p*-values provide some indication of the significance of the observed relationships, these results should be interpreted with caution. The small sample size increases the uncertainty around the estimates, and the findings may not generalize to a broader set of countries. Therefore, the observed trends should be considered indicative rather than conclusive, reflecting potential associations rather than definitive causal relationships.

In analyzing the entropy of COVID-19 hospitalizations and ICU admissions, we also considered health expenditure per capita. Countries with higher spending, such as Switzerland, Austria, and Sweden, generally show lower entropy, suggesting more stable and predictable healthcare responses during the pandemic. In contrast, Czechia and Slovakia, which have lower health expenditures, exhibit the highest entropy values, indicating greater variability in hospital and ICU usage. These results suggest that better-funded healthcare systems may be more resilient and capable of managing pandemic-related fluctuations more effectively. This highlights the role of health investment in promoting system stability and preparedness for future crises.

In the second part of our article, we applied principal component analysis for functional data, obtaining interesting conclusions regarding the training group. Among the eight selected European countries, Switzerland and Portugal had the lowest numbers of hospitalized and ICU patients, and Slovakia and France had the highest. We tested a multiple function-on-function linear model, which effectively predicted the numbers of hospitalized and ICU patients in the training group and for Italy in the testing group. We obtained the worst results for Czechia from the testing group and for Portugal from the training group.

## 10. Discussion and Limitations

This study has several limitations. First, the model was trained on eight European countries and tested only on the Czech Republic and Italy, which may limit the generalizability of the results to other countries with different reporting systems or epidemic dynamics. Second, the analysis covers a relatively short period (7 February to 31 May 2021), and the model may not perform equally well under different pandemic phases or with new viral variants. Third, although multiple performance metrics are reported (MSE and RMSE), the model does not capture all aspects of predictive accuracy, particularly sudden spikes or outliers. Fourth, the model uses only case counts, hospitalizations, deaths, and ICU numbers and does not account for other relevant factors, such as vaccination rates, policy interventions, or demographic differences. Finally, the data quality may vary across countries due to reporting delays or differences in definitions, which could affect the accuracy of the predictions.

The predictive performance of the model varied across countries. For Switzerland and Italy, the predictions were relatively accurate, while, in Czechia and Portugal, the errors were larger. This variability may be related to differences in data reliability and reporting, national healthcare policies influencing hospitalization and ICU admission criteria, and heterogeneity in epidemic dynamics. In addition, the use of a linear model may have limited the ability to capture more complex nonlinear trends. These factors highlight the challenges of cross-country prediction and suggest that future work could explore more flexible modeling approaches.

While in this study we primarily interpreted higher Shannon entropy as an indicator of temporal instability in epidemic dynamics, it is important to note that other explanations are also possible. Elevated entropy values may partly reflect differences in reporting practices, such as delays, data aggregation, or changes in methodology. They may also be influenced by healthcare policies, such as variations in admission criteria for hospitalization or ICU care across countries. Finally, regional outbreak dynamics, including localized surges or heterogeneous transmission patterns, may contribute to greater variability in the data. Considering these factors provides a more comprehensive understanding of entropy as a measure of epidemic complexity.

## Figures and Tables

**Table 1:** Pearson correlation between healthcare expenditure per capita and selected COVID-19 indicators, including *p*-values and 95% confidence intervals.

Variable	Pearson *r*	*p*-Value	95% CI
Confirmed Cases	−0.2506	0.485	[−0.760, 0.450]
Deaths	−0.7341	0.016	[−0.933, −0.194]
Hospitalizations	−0.5217	0.122	[−0.867, 0.161]
ICU Admissions	−0.4974	0.144	[−0.858, 0.193]

**Table 2:** Pearson correlation between healthcare expenditure per capita and confirmed cases and deaths, with addition of Poland. The *t*-statistic and *p*-value test whether the correlation is statistically significant.

Variable	Pearson Coefficient	*t*-Statistic	*p*-Value
Confirmed Cases	−0.1218	−0.368	0.72
Deaths	−0.5919	−2.20	0.054

**Table 3:** Correlation between health expenditure per capita and COVID-19 deaths (with Poland; *n* = 11).

Year	Pearson *r*	*t*-Statistic	*p*-Value
2017	−0.6576	−2.61	0.028
2021	−0.5919	−2.20	0.054
2023	−0.6388	−2.45	0.036

**Table 4:** Correlation between health expenditure per capita and COVID-19 confirmed cases (with Poland; *n* = 11).

Year	Pearson *r*	*t*-Statistic	*p*-Value
2017	−0.2111	−0.662	0.53
2021	−0.1218	−0.368	0.72
2023	−0.1690	−0.523	0.61

**Table 5:** Correlation coefficients between health expenditure per capita and COVID-19 hospitalizations and ICU cases (without Poland). The correlation is not statistically significant, 0.092 < *p* < 0.139.

Year	Hospitalizations vs. Health Expenditure	ICU Cases vs. Health Expenditure
2017	−0.52166	−0.49744
2021	−0.54574	−0.55545
2022	−0.54441	−0.54753

**Table 6:** Shannon entropy of COVID-19-related hospitalizations by country. Higher entropy indicates greater variability in hospitalization patterns.

Country	Hospitalization Entropy (Bits)
Czechia	6.69
Slovakia	6.65
Italy	6.44
Austria	6.34
Slovenia	6.14
Spain	6.07
France	6.06
Portugal	6.03
Sweden	5.63
Switzerland	5.47

**Table 7:** Shannon entropy of COVID-19-related ICU admissions by country. Lower entropy values suggest more consistent ICU usage patterns.

Country	ICU Entropy (Bits)
Czechia	6.15
Slovakia	5.87
Austria	5.24
Spain	5.06
Italy	5.06
France	5.05
Portugal	5.02
Slovenia	5.02
Sweden	4.00
Switzerland	3.60

**Table 8:** Mean squared error for $y_{i1}$ (the number of hospitalized people) and $y_{i2}$ (ICU patients) for the countries from the training group. The largest and smallest values are highlighted in bold.

Country	MSE($y_{i1}$)	MSE($y_{i2}$)
Austria	66.00854	12.943779
France	136.23701	18.949102
Portugal	**146.68951**	**29.428459**
Slovakia	106.56990	19.132264
Slovenia	45.22644	13.204239
Spain	123.58643	15.230891
Sweden	77.75449	12.082737
Switzerland	**37.24373**	**6.752718**

**Table 9:** Root mean square error (RMSE) for $y_{i1}$ (the number of hospitalized people) and $y_{i2}$ (ICU patients) for the countries from the training group. The largest and smallest values are highlighted in bold.

Country	RMSE($y_{i1}$)	RMSE($y_{i2}$)
Austria	8.124	3.598
France	11.675	4.352
Portugal	**12.111**	**5.424**
Slovakia	10.325	4.374
Slovenia	6.728	3.634
Spain	11.117	3.904
Sweden	8.821	3.476
Switzerland	**6.101**	**2.597**

**Table 10:** Selected dates and values of prediction and daily observations of hospitalized patients for testing group.

Date	Obs.—Czechia	Pred.—Czechia	Obs.—Italy	Pred.—Italy
7 February 2021	551.84	325.67614	362.024	522.3150
8 February 2021	593.673	310.91295	367.055	522.2126
9 February 2021	586.336	296.89724	366.801	523.0886
10 February 2021	579.475	283.61196	362.617	524.8128
11 February 2021	577.473	271.04008	356.858	527.2552
12 February 2021	571.375	259.16455	352.844	530.2856
13 February 2021	540.976	247.96834	348.287	533.7738
14 February 2021	561.178	237.43441	347.813	537.5898
15 February 2021	608.634	227.54572	348.999	541.6035
16 February 2021	611.779	218.28524	347.864	545.6846
21 May 2021	84.239	92.47821	344.137	197.4960
22 May 2021	82.428	88.78104	184.933	190.1645
23 May 2021	85.477	85.25691	179.056	183.0197
24 May 2021	76.615	81.94027	175.007	176.1027
25 May 2021	73.947	78.86556	167.351	169.4545
26 May 2021	64.799	76.06719	159.153	163.1160
27 May 2021	59.653	73.57961	150.972	157.1285
28 May 2021	49.838	71.43725	141.165	151.5328
29 May 2021	49.647	69.67455	133.729	146.3700
31 May 2021	53.269	68.32594	129.613	141.6812

**Table 11:** Selected dates and values of prediction and daily observations of ICU patients for testing group.

Date	Obs.—Czechia	Pred.—Czechia	Obs.—Italy	Pred.—Italy
7 February 2021	104.631	55.10461	35.689	66.25172
8 February 2021	105.489	54.43146	36.299	66.74053
9 February 2021	104.25	53.68711	36.299	67.26195
10 February 2021	105.775	52.88075	36.045	67.81437
11 February 2021	106.347	52.02157	36.011	68.39620
12 February 2021	106.156	51.11876	35.486	69.00582
13 February 2021	105.489	50.18153	34.927	69.64163
14 February 2021	109.301	49.21906	35.317	70.30204
15 February 2021	111.874	48.24056	35.384	70.98545
16 February 2021	115.971	47.25521	35.13	71.69024
21 May 2021	18.201	26.00600	24.222	48.61480
22 May 2021	18.011	25.11214	23.883	46.82245
23 May 2021	17.629	24.24109	23.409	45.06300
24 May 2021	15.628	23.39914	22.409	43.34544
25 May 2021	14.866	22.59254	21.647	41.67879
26 May 2021	14.008	21.82758	20.428	40.07206
27 May 2021	12.102	21.11053	19.344	38.53426
28 May 2021	10.768	20.44765	18.548	37.07440
29 May 2021	10.768	19.84524	17.972	35.70149
31 May 2021	11.245	19.30955	17.497	34.42454

## Data Availability

Data are available at https://ourworldindata.org/coronavirus (accessed on 5 June 2025), https://ourworldindata.org/grapher/annual-healthcare-expenditure-per-capita (accessed on 5 June 2025) and https://data.worldbank.org/indicator/SH.XPD.CHEX.PC.CD (accessed on 18 June 2025).
